# The jPOST environment: an integrated proteomics data repository and database

**DOI:** 10.1093/nar/gky899

**Published:** 2018-10-08

**Authors:** Yuki Moriya, Shin Kawano, Shujiro Okuda, Yu Watanabe, Masaki Matsumoto, Tomoyo Takami, Daiki Kobayashi, Yoshinori Yamanouchi, Norie Araki, Akiyasu C Yoshizawa, Tsuyoshi Tabata, Mio Iwasaki, Naoyuki Sugiyama, Satoshi Tanaka, Susumu Goto, Yasushi Ishihama

**Affiliations:** 1 Database Center for Life Science, Joint Support-Center for Data Science Research, Research Organization of Information and Systems, Kashiwa 277-0871, Japan; 2 Niigata University Graduate School of Medical and Dental Sciences, Niigata 951-8510, Japan; 3 Medical Institute of Bioregulation, Kyushu University, Fukuoka 812-8582, Japan; 4 Graduate School of Medical Sciences, Faculty of Life Sciences, Kumamoto University, Kumamoto 860-8556, Japan; 5 Kumamoto University Hospital, Kumamoto 860-8556, Japan; 6 Graduate School of Pharmaceutical Sciences, Kyoto University, Kyoto 606-8501, Japan; 7 Center for iPS Cell Research and Application, Kyoto University, Kyoto 606-8507, Japan; 8 Trans-IT, Kaminokawa 329-0607, Japan

## Abstract

Rapid progress is being made in mass spectrometry (MS)-based proteomics, yielding an increasing number of larger datasets with higher quality and higher throughput. To integrate proteomics datasets generated from various projects and institutions, we launched a project named jPOST (Japan ProteOme STandard Repository/Database, https://jpostdb.org/) in 2015. Its proteomics data repository, jPOSTrepo, began operations in 2016 and has accepted more than 10 TB of MS-based proteomics datasets in the past two years. In addition, we have developed a new proteomics database named jPOSTdb in which the published raw datasets in jPOSTrepo are reanalyzed using standardized protocol. jPOSTdb provides viewers showing the frequency of detected post-translational modifications, the co-occurrence of phosphorylation sites on a peptide and peptide sharing among proteoforms. jPOSTdb also provides basic statistical analysis tools to compare proteomics datasets.

## INTRODUCTION

Proteomics approaches such as mass spectrometry (MS), gel electrophoresis and antibody-based ones are important for identifying proteins with accompanying information such as isoforms, subcellular localizations, tissue specificity, protein interactions, abundances and post-translational modifications (PTMs), many of which cannot be obtained by genomic and transcriptomic approaches. Recent advances in MS technology have significantly improved the coverage, resolution and speed in the measurements and resulted in increasing amounts of larger proteomics data with higher quality and higher throughput. These substantial proteomics data are useful for finding biomarkers in the fields of life and medical sciences, and should be accumulated, published and shared for further analyses (1,2). In fact, proteomics data including MS raw data, peak lists, peptide and protein identification results, and metadata about experiments should be deposited in a public repository when submitting papers describing and using the data, the same as the situation for nucleotide sequence data.

In 2016, we launched a proteomics data repository in Japan, named jPOSTrepo (https://repository.jpostdb.org/) (3), which conforms to the international standards provided by the ProteomeXchange (PX) consortium (4). Currently, jPOSTrepo accepts MS-based proteomics data from all over the world as an official member of the PX consortium, together with PRIDE in Europe (5), MassIVE (https://massive.ucsd.edu/), PASSEL (6) and Panorama Public (7) in U.S.A. and iProX (http://www.iprox.org/) in China. jPOSTrepo has unique features such as a high-speed file upload system and user-friendly interface with open-source libraries; all of the submission operations can be completed within a web browser (3).

Meanwhile, since 2010, the Human Proteome Project of the Human Proteome Organization has been constructing a human proteome database to integrate and organise information on all human proteins and their functions, such as isoforms, temporal variations, localizations, and modifications, although this has yet to be completed (8,9). Although the Human Proteome Map (10) and the ProteomicsDB (11,12) were published as MS-based human proteome databases in 2014, it has been pointed out that they contained many false positives because they detected proteins by combining spectra from multiple experiments containing many low-quality spectra (13). As such, we should develop a high-quality proteome database to obtain highly accurate information of proteoforms with few false positives from MS raw files.

Here, we present the current status of the jPOST environment; updates of jPOSTrepo in the past two years and a newly developed proteome database, named jPOSTdb (https://globe.jpostdb.org/), containing standardized proteomics data reanalyzed from the published raw data in the jPOSTrepo with a common protocol and under the same confidence criteria (14). These data are also well curated with experimental metadata based on controlled selections of biological ontologies. jPOSTdb provides graphical data visualization interfaces for identified proteins, including protein annotations, identified peptide mapping, frequencies of detected PTMs, co-occurrence of phosphorylation sites on a peptide and peptide sharing among isoforms and among proteins. To the best of our knowledge, the interface for visualizing quantitative data and co-occurrence information of PTMs and peptide sharing is a unique feature of jPOSTdb.

## CURRENT STATUS AND UPDATES OF REPOSITORY

**Figure 1.**
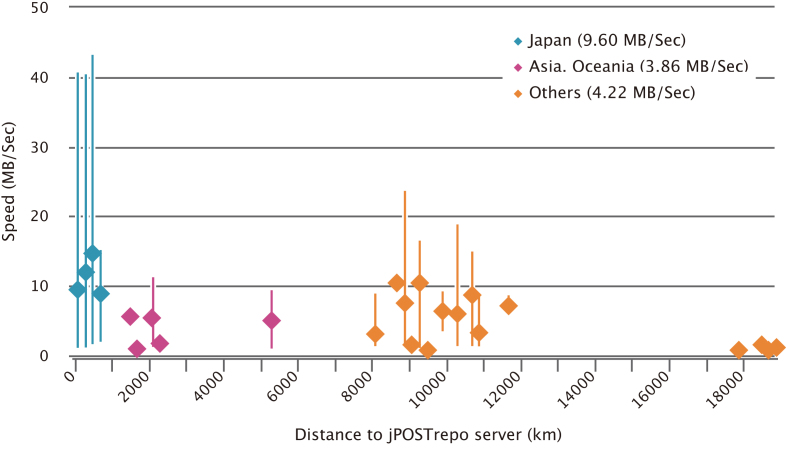
File upload speed to jPOSTrepo. Each square represents the mean upload speed at every 200 km from each place to jPSOTrepo server installed at Database Center for Life Science in Mishima, Japan and the corresponding bar shows maximum speed and minimum speed.

jPOSTrepo was launched in May 2016 and joined the PX consortium in July, 2016. Currently, jPOSTrepo has 320 projects (170 public) with more than 10TB of data; this number of projects is threefold compared with that of 2 years ago, whereas the amount of data has increased 7-fold. The datasets have been submitted from 20 countries, including not only those in Asia and Oceania, as originally expected, but also other countries in Europe, North and South America and elsewhere. One of the key features of jPOSTrepo is that it allows the rapid uploading of files. Although it depends on the local network environment, the average file upload speed is 9.60MB/s from Japan, 3.86MB/s from elsewhere in Asia and Oceania, and 4.22MB/s from other areas (Figure 1). This means that it takes only 1 h at most to upload a 10GB file on an average.

In addition to MS data, jPOSTrepo now accepts gel electrophoresis-based proteomics data (such as those from 2D-PAGE and 2D-DIGE) and antibody-based proteomics data, covering almost all types of proteomics data. On the submission page, users can select which type of dataset to submit. Because the PX consortium accepts only MS data, datasets of other data types cannot be assigned a PX identifier. However, jPOSTrepo issues a jPOST identifier for non-MS datasets; thus, users are able to use a jPOST identifier instead of a PX identifier.

## OVERVIEW OF DATABASE

The back-end system of jPOSTdb supports not only human but also a wide range of species such as model animals, plants, yeasts, and bacteria, unlike existing databases targeted only to human. The current version of the database contains reanalyzed datasets from samples of human, mouse, rat and bacteria selected by checking metadata and raw data quality. For the metadata, information on the sample source, sample preparation method and condition of mass spectrometry acquisition is required. Each piece of metadata is curated manually for each dataset based on various ontologies of the life science field, such as National Center for Biotechnology Information taxonomy (15), Cell Line Ontology (16), Braunschweig Enzyme Database Tissue and Enzyme Source Ontology (17), Human Disease Ontology (18), National Cancer Institute Thesaurus (19) and the Human Proteome Organization Proteomics Standards Initiative-Mass Spectrometry controlled vocabulary (20). We inspected the raw data quality based on the following factors: (i) the peptide-spectrum match (%) should be >10%, (ii) LC chromatograms should not have irregular peak profiles and (iii) the distribution of delta mass for precursor ions should follow the Gaussian pattern.

jPOSTdb stores assigned peptide-spectrum matches (PSMs), identified peptides, inferred proteins and dataset information hierarchically. For peptide identification, jPOSTdb uses the UniProt ‘proteomes’, which are the protein sequence set thought to be expressed by an organism whose genome has been completely sequenced (21). Proteins are inferred based on identified peptides by using the method proposed by Nesvizhskii and Aebersold (22). In jPOSTdb, the entire dataset as a single unit is named a ‘Globe’, and datasets selected from the Globe by users with their own filters is named a ‘Slice’. jPOSTdb provides the following functions: (i) datasets filtering by metadata, to create a ‘Slice’; (ii) browsing identified peptides, PTMs and other data and (iii) basic statistical analysis and visualization of data in specified ‘Slices’.

**Figure 2.**
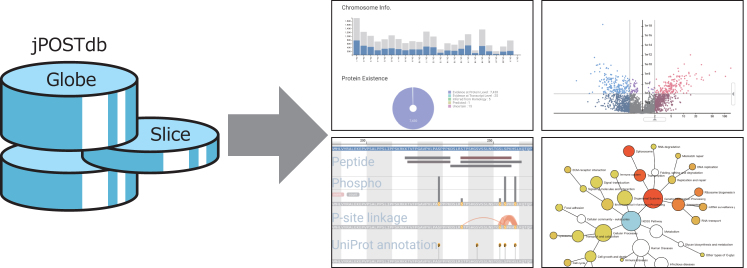
Overview of the jPOSTdb web interface. ‘Globe’ is the whole data set of jPOSTdb, whereas ‘Slice’ is datasets filtered by metadata. Details of the example views on the right are described in Figures 3–5.

Figure 2 shows an overview of the jPOSTdb web interface. First, users can filter a ‘Globe’ by a faceted search based on a combination of dataset metadata such as species, sample type, cell line, organ, disease, modifications and MS instruments, as well as a simple keyword search. Then, users can save the filtered dataset to a ‘Slice’, which means a personalized sub-dataset of a ‘Globe’, from the checkboxes of the results table and ‘Add to Slice’ button. Because the ‘Slice’ data are stored in the web storage of the user's browser, a saved ‘Slice’ is not sent to the server or any other location at all, and the different browsers present on a computer do not share data of web storage. Users thus need to export and import the ‘Slice’ to observe the same datasets in different locations. After the dataset filtering, users can access the detected peptides, PTMs, inferred proteins and other proteomics information. Additionally, users can perform comparative analyses between ‘Slices’ by using the jPOSTdb which implements basic statistical and functional analyses, such as differential expression analysis and enrichment analysis. The details of this are described below.

## INTERFACES OF DATABASE

**Figure 3.**
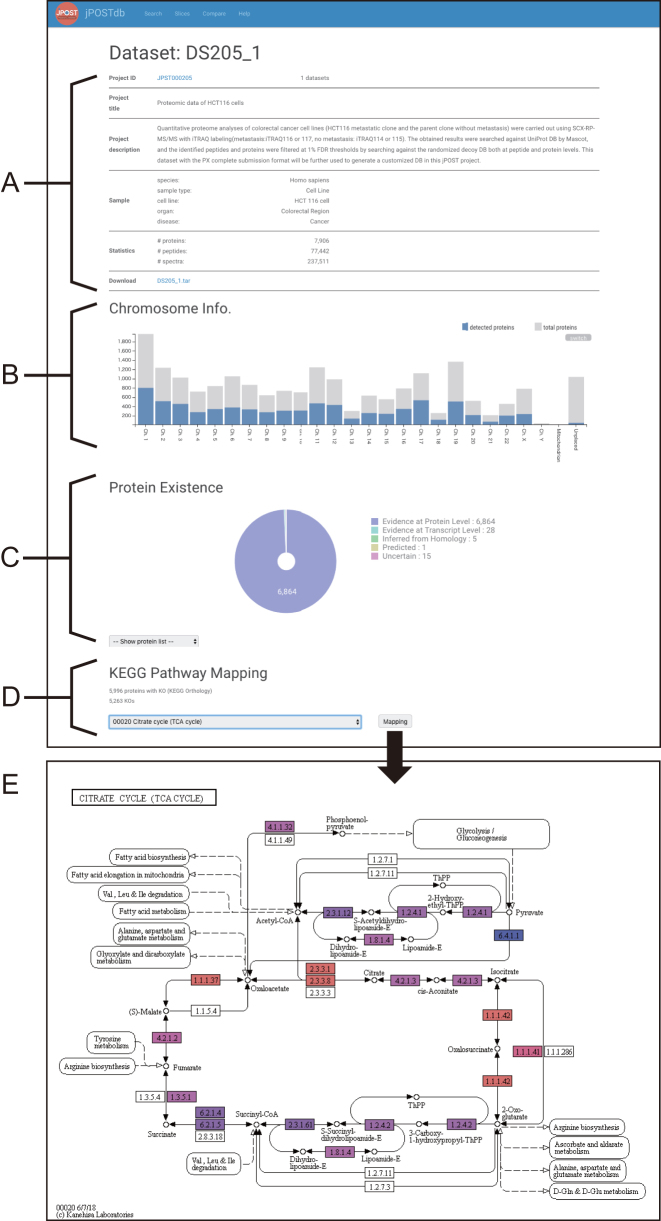
Example of dataset entry page. (**A**) Metadata and statistics of dataset. (**B**) Histogram of proteins per chromosome. Chromosome annotations are based on UniProt, where ‘unplaced’ means chromosome information is not available. (**C**) Pie chart of protein existence. (**D**) Input form for KEGG pathway mapping. (**E**) Example of KEGG pathway mapping. The protein box color varies from red to blue, corresponding to high and low expression, respectively, based on their spectral counts.

**Figure 4.**
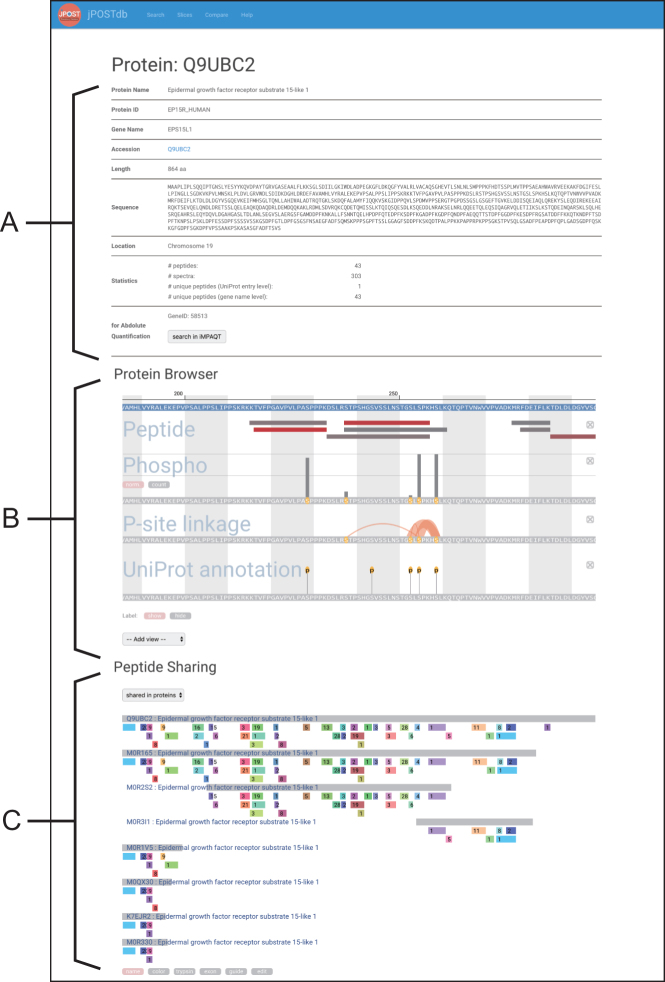
Example of protein entry page. (**A**) Metadata and statistics of a protein. (**B**) Viewer of protein annotations. (**C**) Viewer of peptide sharing among isoforms and proteins. Peptide bars with the same color indicate the same peptides.

jPOSTdb is organized by the following three types of entry: dataset entry, protein entry and peptide entry. Each entry page contains a data summary and lists of corresponding proteins, peptides and PSMs. In the dataset and protein entry pages, jPOSTdb provides graphical data visualization interfaces (Figures 3 and 4). In addition, jPOSTdb also provides a ‘Slice’ page with graphical interfaces as the dataset page. Users can download the data tables of PSMs and inferred proteins through the corresponding dataset entry page and the jPOST FTP site (ftp://jpost.pharm.kyoto-u.ac.jp/database/).

### Dataset entry page

#### Chromosome info

The ‘Chromosome info’ section shows a histogram of detected proteins (blue) and the total number of proteins (grey) per chromosome (Figure 3B), mitochondria and plasmids listed on the dataset entry page and slice page. The protein count is calculated based on the protein entry number in UniProt (15). Therefore, the total number of proteins does not refer to the exact number of coding genes in each chromosome. In cases in which the species of a dataset/slice is human, the protein count is based on the count of neXtProt entries (23).

#### Protein existence

The ‘Protein existence’ section displays a pie chart that shows the evidence types that support the existence of proteins (Figure 3C), described in the neXtProt database for human and the UniProt database for other organisms. There are five types of evidence for the existence of a protein as follows: PE1) experimental evidence at the protein level, PE2) experimental evidence at the transcript level, PE3) protein inferred from homology, PE4) protein predicted and PE5) protein uncertain, in that there is uncertainty over whether the protein actually exists. In case of human, proteins in PE2–4 categories are called ‘missing proteins’, indicating that they are unconfirmed sequences for which protein products have not yet been detected; the detection of these proteins has been the focus of chromosome-centric human proteome project (9).

#### KEGG pathway mapping

Proteins with KEGG Orthology (KO) annotation can be mapped to KEGG PATHWAY (24) (Figure 3D). The protein box color on KEGG pathway maps varies from red to blue, corresponding to high and low expression, respectively, based on their spectral counts.

### Protein entry page

#### Protein browser

The ‘Protein browser’ section is a viewer of protein structural annotations (Figure 4B). Users can add annotations of interest into the viewer panel from the ‘Add view’ pull-down menu. The ‘Peptide’ panel shows detected peptides mapped to the protein sequence. The color of peptide bars reflects the number of PSMs, and varies from red to grey (red represents a high number and grey represents a low one). The ‘PTM’ panel displays actual modification names such as ‘Phospho’ in the viewer, and shows detected PTMs on the protein sequence. The vertical bar length above the PTM site reflects the count of PTM detection. Here ‘norm’. represents the normalized length by spectral counts included in the site, and ‘count’ shows real counts. The ‘P-site linkage’ panel shows the co-occurrence of phosphorylation sites on a peptide. To the best of our knowledge, the comprehensive PTM counting and PTM co-occurrence are unique features of jPOSTdb. The ‘UniProt annotation’ panel shows PTM sites and single amino acid variations described in UniProt.

#### Peptide sharing

The ‘Peptide sharing’ section shows peptides with the same amino acid sequence mapping to multiple UniProt isoforms and multiple UniProt entries, separately (Figure 4C). Peptide bars with the same color across multiple sequences indicate the same peptides. The numerical values overlapped with peptide bars refer to the number of PSMs. In MS-based shotgun proteomics, instead of full-length proteins, the peptides digested by enzymes such as trypsin are detected. Therefore, it is important to clarify which protein is more likely to be the origin of each peptide for the inference of protein expression. On this interface, users can visualize which proteins share peptides with each other. Users can also recognize whether the peptide of interest has tryptic cleavage sites at both termini.

## COMPARISON OF SELECTED DATASETS

Because jPOSTdb provides basic statistical analysis tools, users can compare the statistics and expression of proteins between ‘Slices’.

### Differential expression analysis

**Figure 5.**
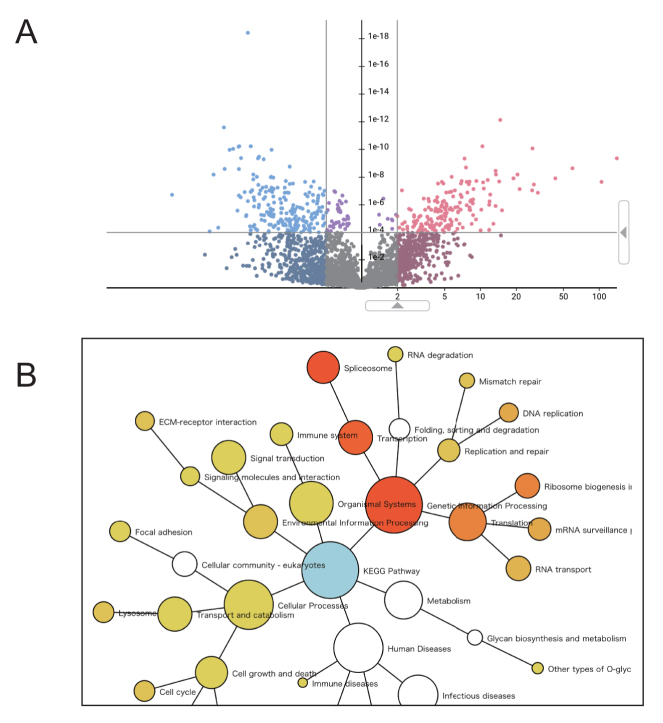
Example of comparison between ‘Slices’. (**A**) Result example of differential expression analysis. (**B**) Network graph of enrichment analysis (in the KEGG pathway category).

Users can compare the expression level of proteins between two ‘Slices’ by empirical Bayes estimate, Wilcoxon rank sum test and fold change of the mean from the pull-down menu. This quantification is based on spectral counting normalized by the total count of spectra from each dataset. The former two methods are implemented by using the R programming language library. The Wilcoxon rank sum test is a statistical test commonly used in differential expression analyses when the number of datasets is large enough. The empirical Bayes estimation is applicable even when the number of datasets is relatively small. In the volcano plot of results, users can change thresholds of the fold change and *P*-value by moving the triangular marker on the x- and y-axes (Figure 5A). The fold change of the mean expression level in a protein is shown as a histogram-like plot. It is not subjected to any statistical test, so the *P*-value is not calculated; therefore, the y-axis shows an arbitrary scale based on frequency.

### Enrichment analysis

jPOSTdb provides protein set enrichment analysis targeting the KEGG pathway category and the three main categories of Gene Ontology (GO) (biological process, molecular function, and cellular component) (25), for selected proteins in the differential expression analysis. The results are displayed by a network graph (Figure 5B) and a table. Nodes in the network show categories of the KEGG pathway or the GO, and the node color of enriched categories varies from yellow to red (*P*-value < 0.05, yellow shows high and red shows low). The blue node shows the root category of the network. Each node's size reflects the number of selected proteins in the differential expression analysis. However, the size of white nodes and the root node is limited to the maximum size of enriched nodes that are colored from yellow to red, to make the network layout clearer. When the target is a KEGG pathway, users can map proteins onto the KEGG pathway map. The boxes of pathway maps are colored from blue to red (blue shows that the expression level is decreased and red shows that it is increased). When a box in pathway maps corresponds to multiple proteins, the box is colored arbitrarily by any one of these proteins (due to a limitation of the KEGG mapper).

## DISCUSSION

**Figure 6.**
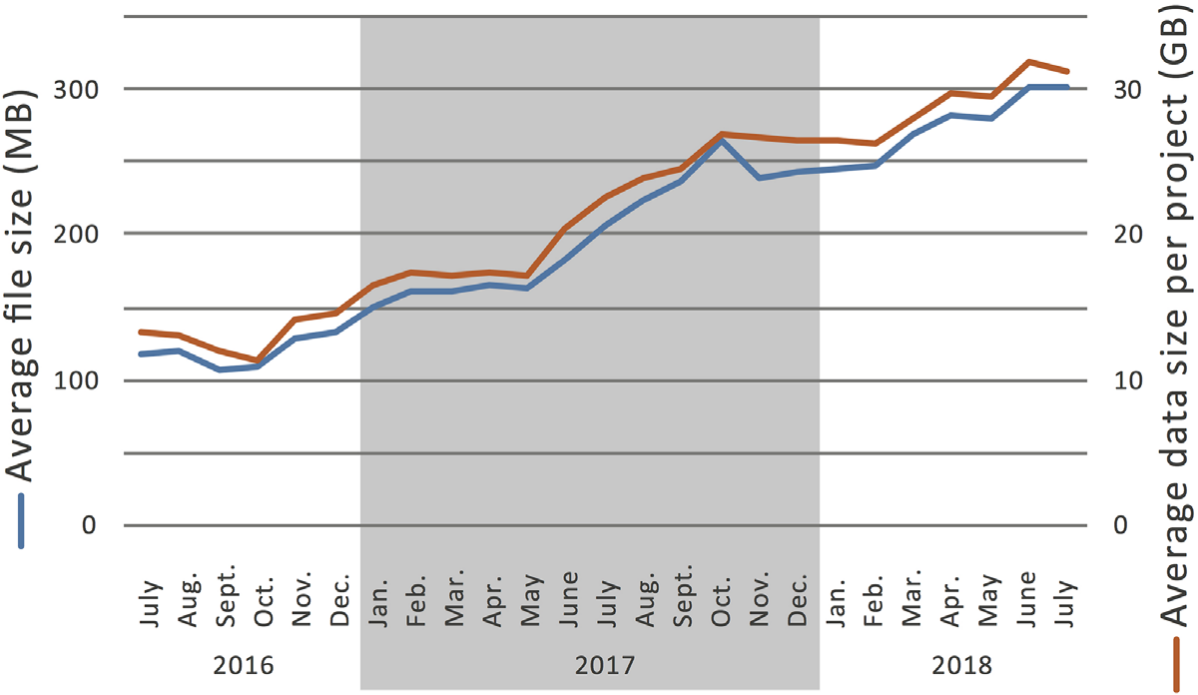
The growing of submitted data size to jPOSTrepo. Blue line shows the average file size (left y-axis), and red line shows the average data size per project (right y-axis).

Increasing number of projects has been continuously deposited in jPOSTrepo from all over the world. In addition, the average data size per project is increasing, whereas the number of files per project is constant, indicating that the average size of an MS raw file is increasing (Figure 6). This would be because of higher resolving power of mass analyzers and higher frequencies to acquire MS/MS spectra in newly launched MS instruments, resulting in higher coverage of proteomes with higher throughput than earlier. In addition, ultra-large-scale proteomics projects using human patient samples have been launched, such as the international cancer proteogenome consortium (https://icpc.cancer.gov/) and the Human Diabetes Proteome Project (26). Hence, it is imperative for the public repository to equip itself with a highly efficient system to upload/download the data, which has been achieved in jPOSTrepo (Figure 1). Furthermore, jPOSTrepo continuously receives the MS-based proteomics data of non-human organisms, as well as the gel-based and the antibody-based proteomics data. Taken together, a wide variety of proteome datasets has been successfully accumulated in jPOSTrepo and is converted into jPOSTdb using the standardized data analysis protocol.

Currently, jPOSTdb automatically calculates spectral counts for quantitative analyses, and uses other quantitative approaches, such as stable isotope labelling for relative and absolute quantitation, including SILAC, dimethyl labelling, iTRAQ and TMT, and label-free quantitation based on extracted ion chromatograms, emPAI (27) and iBAQ (28) as a next step.

jPOSTdb is constructed based on the Semantic Web technology that facilitates integration of various databases containing big data (29). The protein sequence and annotation databases, such as UniProt and neXtProt, have released data formatted by the Resource Description Framework (RDF) data model, which supports the Semantic Web technology (21,30). In addition, not only the proteome data, but also other omics and life science-related data have been converted to RDF data and released in the NBDC RDF portal (https://integbio.jp/rdf/) and EBI RDF platform (31) and have been incorporated into various life science databases such as TogoGenome, a genomic database based on RefSeq data (http://togogenome.org/), TogoVar, a database of human genome variants/variations (https://togovar.biosciencedbc.jp/) and GlyTouCan, a glycan structure repository (32). Based on the RDF format, therefore, data integration as well as database integration will be more accelerated in future, and the high-quality proteome data generated from the jPOST environment would be one of its core elements because proteins are lethally essential biomolecules for the functioning of cells.

## References

[1] Perez-Riverol Y., Alpi E., Wang R., Hermjakob H., Vizcaíno J.A. Making proteomics data accessible and reusable: current state of proteomics databases and repositories. Proteomics. 2015; 15:930–949. 25158685 10.1002/pmic.201400302 PMC4409848

[2] Martens L., Vizcaíno J.A. A golden age for working with public proteomics data. Trends Biochem. Sci. 2017; 42:333–341. 28118949 10.1016/j.tibs.2017.01.001 PMC5414595

[3] Okuda S., Watanabe Y., Moriya Y., Kawano S., Yamamoto T., Matsumoto M., Takami T., Kobayashi D., Araki N., Yoshizawa A.C. jPOSTrepo: an international standard data repository for proteomes. Nucleic Acids Res. 2017; 45:D1107–D1111. 27899654 10.1093/nar/gkw1080 PMC5210561

[4] Deutsch E.W., Csordas A., Sun Z., Jarnuczak A., Perez-Riverol Y., Ternent T., Campbell D.S., Bernal-Llinares M., Okuda S., Kawano S. The ProteomeXchange consortium in 2017: supporting the cultural change in proteomics public data deposition. Nucleic Acids Res. 2017; 45:D1100–D1106. 27924013 10.1093/nar/gkw936 PMC5210636

[5] Vizcaíno J.A., Csordas A., Del-Toro N., Dianes J.A., Griss J., Lavidas I., Mayer G., Perez-Riverol Y., Reisinger F., Ternent T. 2016 update of the PRIDE database and its related tools. Nucleic Acids Res. 2016; 44:D447–D456. 26527722 10.1093/nar/gkv1145 PMC4702828

[6] Farrah T., Deutsch E.W., Kreisberg R., Sun Z., Campbell D.S., Mendoza L., Kusebauch U., Brusniak M.Y., Hüttenhain R., Schiess R. PASSEL: the PeptideAtlas SRMexperiment library. Proteomics. 2012; 12:1170–1175. 22318887 10.1002/pmic.201100515 PMC3832291

[7] Sharma V., Eckels J., Schilling B., Ludwig C., Jaffe J.D., MacCoss M.J., MacLean B. Panorama Public: a public repository for quantitative data sets processed in Skyline. Mol. Cell. Proteomics. 2018; 17:1239–1244. 29487113 10.1074/mcp.RA117.000543 PMC5986241

[8] Legrain P., Aebersold R., Archakov A., Bairoch A., Bala K., Beretta L., Bergeron J., Borchers C.H., Corthals G.L., Costello C.E. The human proteome project: current state and future direction. Mol. Cell. Proteomics. 2011; 10:doi:10.1074/mcp.M111.009993. 10.1074/mcp.M111.009993 PMC3134076 21742803

[9] Omenn G.S., Lane L., Lundberg E.K., Overall C.M., Deutsch E.W. Progress on the HUPO draft human Proteome: 2017 metrics of the human proteome project. J. Proteome Res. 2017; 16:4281–4287. 28853897 10.1021/acs.jproteome.7b00375 PMC5872831

[10] Kim M.S., Pinto S.M., Getnet D., Nirujogi R.S., Manda S.S., Chaerkady R., Madugundu A.K., Kelkar D.S., Isserlin R., Jain S. A draft map of the human proteome. Nature. 2014; 509:575–581. 24870542 10.1038/nature13302 PMC4403737

[11] Wilhelm M., Schlegl J., Hahne H., Gholami A.M., Lieberenz M., Savitski M.M., Ziegler E., Butzmann L., Gessulat S., Marx H. Mass-spectrometry-based draft of the human proteome. Nature. 2014; 509:582–587. 24870543 10.1038/nature13319

[12] Schmidt T., Samaras P., Frejno M., Gessulat S., Barnert M., Kienegger H., Krcmar H., Schlegl J., Ehrlich H.C., Aiche S. PoteomicsDB. Nucleic Acids Res. 2018; 46:D1271–D1281. 29106664 10.1093/nar/gkx1029 PMC5753189

[13] Ezkurdia I., Vázquez J., Valencia A., Tress M. Analyzing the first drafts of the human proteome. J. Proteome Res. 2014; 13:3854–3855. 25014353 10.1021/pr500572z PMC4334283

[14] Yoshizawa A.C., Tabata T., Iwasaki M., Sugiyama N., Ishihama Y. Utilizing peptide sequence tags for controlling false discovery rates in database search. Proceedings of the 66th ASMS Conference on Mass Spectrometry and Allied Topics, ThP415. 2018.

[15] Federhen S. The NCBI taxonomy database. Nucleic Acids Res. 2012; 40:D136–D143. 22139910 10.1093/nar/gkr1178 PMC3245000

[16] Sarntivijai S., Lin Y., Xiang Z., Meehan T.F., Diehl A.D., Vempati U.D., Schürer S.C., Pang C., Malone J., Parkinson H. CLO: the cell line ontology. J. Biomed. Semantics. 2014; 5:37. 25852852 10.1186/2041-1480-5-37 PMC4387853

[17] Gremse M., Chang A., Schomburg I., Grote A., Scheer M., Ebeling C., Schomburg D. The BRENDA Tissue Ontology (BTO): the first all-integrating ontology of all organisms for enzyme sources. Nucleic Acids Res. 2011; 39:D507–D513. 21030441 10.1093/nar/gkq968 PMC3013802

[18] Kibbe W.A., Arze C., Felix V., Mitraka E., Bolton E., Fu G., Mungall C.J., Binder J.X., Malone J., Vasant D. Disease Ontology 2015 update: an expanded and updated database of human diseases for linking biomedical knowledge through disease data. Nucleic Acids Res. 2015; 43:D1071–D1078. 25348409 10.1093/nar/gku1011 PMC4383880

[19] Noy N.F., de Coronado S., Solbrig H., Fragoso G., Hartel F.W., Musen M.A. Representing the NCI Thesaurus in OWL DL: Modeling tools help modeling languages. Appl. Ontol. 2008; 3:173–190. 19789731 10.3233/AO-2008-0051 PMC2753293

[20] Mayer G., Montecchi-Palazzi L., Ovelleiro D., Jones A.R., Binz P.A., Deutsch E.W., Chambers M., Kallhardt M., Levander F., Shofstahl J. HUPO-PSI Group. The HUPO proteomics standards initiative- mass spectrometry controlled vocabulary. Database. 2013; 2013:bat009. 23482073 10.1093/database/bat009 PMC3594986

[21] The UniProt Consortium UniProt: the universal protein knowledgebase. Nucleic Acids Res. 2017; 45:D158–D169. 27899622 10.1093/nar/gkw1099 PMC5210571

[22] Nesvizhskii A.I., Aebersold R. Interpretation of shotgun proteomic data the protein inference problem. Mol. Cell. Proteomics. 2005; 4:1419–1440. 16009968 10.1074/mcp.R500012-MCP200

[23] Gaudet P., Michel P.A., Zahn-Zabal M., Cusin I., Duek P.D., Evalet O., Gateau A., Gleizes A., Pereira M., Teixeira D. The neXtProt knowledgebase on human proteins: current status. Nucleic Acids Res. 2015; 43:D764–D770. 25593349 10.1093/nar/gku1178 PMC4383972

[24] Kanehisa M., Furumichi M., Tanabe M., Sato Y., Morishima K. KEGG: new perspectives on genomes, pathways, diseases and drugs. Nucleic Acids Res. 2017; 45:D353–D361. 27899662 10.1093/nar/gkw1092 PMC5210567

[25] Gene Ontology Consortium Gene Ontology Consortium: going forward. Nucleic Acids Res. 2015; 43:D1049–D1056. 25428369 10.1093/nar/gku1179 PMC4383973

[26] Schvartz D., Bergsten P., Baek K.H., Barba De La Rosa A., Cantley J., Dayon L., Finamore F., Fontana P., Gaudet P., Goo Y.A. The Human Diabetes Proteome Project (HDPP): the 2014 update. Transl. Proteomics. 2015; 8:1–7.

[27] Ishihama Y., Oda Y., Tabata T., Sato T., Nagasu T., Rappsilber J., Mann M. Exponentially modified protein abundance index (emPAI) for estimation of absolute protein amount in proteomics by the number of sequenced peptides per protein. Mol. Cell. Proteomics. 2005; 4:1265–1272. 15958392 10.1074/mcp.M500061-MCP200

[28] Schwanhäusser B., Busse D., Li N., Dittmar G., Schuchhardt J., Wolf J., Chen W., Selbach M. Global quantification of mammalian gene expression control. Nature. 2011; 473:337–342. 21593866 10.1038/nature10098

[29] Wu H., Yamaguchi A. Semantic Web technologies for the big data in life sciences. Biosci. Trends. 2014; 8:192–201. 25224624 10.5582/bst.2014.01048

[30] Chichester C., Karch O., Gaudet P., Lane L., Mons B., Bairoch A. Converting neXtProt into Linked Data and nanopublications. Semantic Web. 2015; 6:147–153.

[31] Jupp S., Malone J., Bolleman J., Brandizi M., Davies M., Garcia L., Gaulton A., Gehant S., Laibe C., Redaschi N. The EBI RDF platform: linked open data for the life sciences. Bioinformatics. 2014; 30:1338–1339. 24413672 10.1093/bioinformatics/btt765 PMC3998127

[32] Tiemeyer M., Aoki K., Paulson J., Cummings R.D., York W.S., Karlsson N.G., Lisacek F., Packer N.H., Campbell M.P., Aoki N.P. GlyTouCan: an accessible glycan structure repository. Glycobiology. 2017; 27:915–919. 28922742 10.1093/glycob/cwx066 PMC5881658

